# Action‐selection perseveration in young children: Advances of a dynamic model

**DOI:** 10.1002/dev.21776

**Published:** 2018-09-16

**Authors:** Ralf F. A. Cox, Ad W. Smitsman

**Affiliations:** ^1^ Department of Psychology University of Groningen Groningen The Netherlands; ^2^ Behavioural Science Institute Radboud University Nijmegen The Netherlands

**Keywords:** action selection, development, dynamic model, handedness, perseveration, tool use

## Abstract

This study presents an empirical test and dynamic model of perseverative limb selection in children of 14‐, 24‐, and 36‐months old (*N* = 66 in total). In the experiment, children repeatedly grasped a spoon with a single hand. In two separate conditions, the spoon was presented either four times on their right side or four times on their left side. In both conditions, following this training, the spoon was presented on midline for two more trials. This setup enabled us to determine whether children's limb selection was influenced by their prior choices in the task (i.e., perseveration). Individual children's handedness was determined in a third condition consisting of nine object presentations (laterally or on midline). A dynamic model for limb selection is presented combining external input, motor memory, and preferences. The model was used to simulate the experiment and reproduced the results, including the age‐related changes.

## 1 INTRODUCTION

Studies on goal‐directed behavior in children have shed light on the development of action planning and, more in particular, on the development of action selection. The term action selection highlights the choices for action children have to make when trying to attain a goal. In general, such choices depend on the information that is available with respect to the goal, the means, and task constraints that may affect goal attainment. Action selection as part of goal‐directed behavior has been studied extensively in the context of tool use (e.g., Claxton, McCarty & Keen, 2009; Cox & Smitsman, 2006a,2006b; Jovanovic & Schwarzer, 2017; McCarty, Clifton & Collard, 1999; McCarty, Clifton & Collard, 2001; Smitsman & Cox, 2008). Although we have learned a great deal from these studies with respect to the factors involved in the choices children make, the issue is still unresolved as to how these different factors combine in the underlying action‐selection process. Moreover, we know very little about the development of this process during the first years of life.

Reaching toward a location in hemispace (e.g., for grasping a tool) certainly is a basic example of a goal‐directed action. Reaching and grasping are extensively studied in motor‐development research, and a great deal is known about their movement organization and development (e.g., Corbetta & Thelen, 1996, 2002; Spencer, Vereijken, Diedrich & Thelen, 2000; Thelen et al., 1993; Van Hof, Van der Kamp & Savelsbergh, 2002; Von Hofsten, 1984, 1991). With respect to unimanual reaching and grasping, an essential part of planning such a movement is the choice of which hand to use, generally referred to as limb selection. This study addresses young children's limb selection in unimanual grasping of a simple hand‐held tool. By focusing on limb selection, the action system under study can basically be considered as a bistable system. We propose that the overt behavior of this action system (i.e., the hand that is used) is governed by an underlying action‐selection process. In the following, we will elaborate on the implications of this proposition and on the factors that influence this selection process.

Many of the factors that influence children's limb selection are known, such as, handedness (e.g., Hopkins & Rönnqvist, 1998; Rat‐Fischer, O'Regan & Fagard, 2013), object location (e.g., Gabbard & Rabb Helbig, 2004; Gabbard, Rabb Helbig & Gentry, 2001; Harris & Carlson, 1993; Van Hof et al., 2002), task complexity (e.g., Bryden, Pryde & Roy, 1999; Gonzalez, Flindall & Stone, 2015; Jacquet, Esseily, Rider & Fagard, 2012; Leconte & Fagard, 2004; Sacrey, Arnold, Whishaw & Gonzalez, 2013). It has also been suggested that not one of these factors solely determines the limb that is selected in a particular task, but rather that they combine in the selection process (Cox & Smitsman, 2006b, 2008; Leconte & Fagard, 2004; Smitsman & Cox, 2008). Moreover, the influence of at least some of these factors changes with age. For instance, it is well known that handedness increases in strength during the first 3 years of life and also onward (McManus et al., 1988; Scharoun & Bryden, 2014). This particular combination (i.e., combined influence of factors and age‐related changes) makes limb selection quite interesting for studying action planning from a developmental perspective. Despite the obvious relevance of the subject for understanding (uni‐/bi‐)manual behavior, inter‐limb coordination and, more generally, for gaining insight into the development of action planning, our knowledge about the way reaching and grasping is organized at the level of limb selection still remains limited (Bryden, 2015; Gabbard & Rabb, 2000).

Earlier, Cox and Smitsman (2006b) reported a study on the confluence of factors in young children's choice of what hand to use in a goal‐directed task. In the experiment, 24‐ and 36‐month‐olds had to grasp and subsequently use a hand‐held tool (a cane) with one hand, in order to transport an object toward a goal. Interestingly, the experiments showed that the hand that was selected initially was altered during task performance in some situations but not in others. Children switched the cane from one hand to the other between the two phases of the task (i.e., between grasping and transporting), as a result of specific combinations of the relevant factors (viz. handedness, initial handle orientation, and goal location). This behavior could not be explained in terms of the dominance of a single one of these variables. Stated differently, a number of different factors mutually contributed at the same time in the selection of the limb to perform the task with. Moreover, this demonstrated that limb selection only temporarily favored one hand above the other and that changing information about the relevant variables during the course of the action could either reinforce or weaken earlier choices. In the latter case, this led to switching of the cane between the hands. In the researchers’ opinion, these results revealed that a dynamic process must be governing limb selection.

Generally speaking, action‐selection processes typically involve a mapping between, on the one hand, sensorimotor and intentional information, and, on the other hand, some type of motor output (e.g., a particular limb). Interesting insights on the mechanisms behind this mapping are to be gained in situations where the obvious, natural, or initial choice becomes challenged because of the competition between the different factors involved. An interesting example where this is the case, comes from tasks that extend over a longer period than a mere singleton action. In such tasks, the recent history of the system comes into play, and perseveration might be observed as an overt behavioral phenomenon. Fundamentally, perseveration reveals that the action‐selection process is affected by the history of earlier selections, which (temporarily) overrule the influence of the sensorimotor and intentional information that is available. In this context, perseveration gives us a window on the development of planning, by revealing the multi‐causal and multiple‐timescale dynamics of action selection.

A famous example of perseverative action selection is the A‐not‐B error (Piaget, 1954), which constitutes a cornerstone in our thinking about the development of executive functions and planning: Infants in Piaget's stage IV (approximately 7–12 months of age) sometimes reach erroneously to a location (A), in search of an object. They do so after having retrieved the object on that location a number of times and despite having witnessed the object being hidden at another location (B). This error has been successfully explained and modeled by Thelen, Schöner, Scheier and Smith (2001) using a model that was inspired by a dynamic and embodied view of planning. The model is based on the more general dynamic field theory of movement programming (Erlhagen & Schöner, 2002).

Previously, we used a discrete (i.e., two‐neuron) version of the dynamic field model mentioned above to simulate limb selection in adults (Cox & Smitsman, 2018). The model implemented the limb‐selection process, thereby stressing the multi‐timescale aspects of the underlying dynamics. This enabled us to better understand and quantitatively simulate the effects of perseverance and hysteresis in limb selection, which we found in our experiments. In the model, the choice for a hand to grasp an object with emerges gradually, driven by intertwining real time, embodied processes like (motor) memory, inhibition, perception, and noise. The importance of such an intrinsic dynamics inherent to hand use had been pointed out by others (e.g., Corbetta & Thelen, 1996, 2002; Leconte & Fagard, 2004; Van Hof et al., 2002; see also Carson, 1993).

For a more detailed account of the limb‐selection model, we refer to Cox and Smitsman (2018). Here, it will suffice to recall that a significant feature of the model was the combination of two basic mechanisms that underlie limb selection, viz. limb dominance and attentional information (Gabbard & Rabb, 2000). Attentional information refers to the environmental and task‐related parts of the constraints that influence limb selection (Newell, 1986), and which are generally perceptual in nature. Limb dominance (i.e., handedness) is an organismic constraint (Newell), because it is functionally or possibly even structurally connected to the action system. In this study, we will present an extension of our limb‐selection model, based on empirical findings on the development of handedness. This model will be used to simulate the experiment we performed on young children's perseverative limb selection. Below, we will introduce the setup of the experiment, followed by a discussion on handedness and attentional information, and the way they are involved in the experiment.

In the main part of the experiment, children of 14‐, 24‐, and 36‐months old, grasped for a spoon with one hand and subsequently (pretend to) feed a puppet. In two separate conditions, children received a series of “training” trials, designed to build up a short‐term bias for using one of the hands. In each condition, following this training, there were two “neutral” trials in order to measure the effect of the bias on subsequent limb selections. Specifically, in the training trials, spoon position and handle orientation were either both left or both right, provoking the children to use the left or right hand, respectively. In the follow‐up neutral trials, the spoon was presented on midline with the handle pointing toward the child, that is, neutral with respect to laterality. Because of the identical set of neutral trials, we reasoned that a difference in limb selection after the two training series could only be the result of the short‐term bias. Therefore, if a difference is found, it must be interpreted as a perseveration effect, revealing the influence of the system's recent history. This then suggests that limb selection must be operating on multiple timescales.

Limb selection in children (as well as in adults) is, to a large extent, directly guided by online sensory (attentional) information about the spatial (extrinsic) properties of objects. For instance, McCarty et al. (1999, 2001) demonstrated that in 19‐month‐olds, hand choice in spoon grasping ipsilateraly reflects the orientation of the spoon's handle. Such an effect of orientation has also been demonstrated by Cox and Smitsman (2006a,2006b) for slightly older children in grasping a cane. In addition, other studies have reported a close ipsilateral link between object location and hand choice in grasping (Gabbard & Rabb Helbig, 2004; Gabbard et al., 2001; Harris & Carlson, 1993; Van Hof et al., 2002). More generally, numerous spatial‐compatibility effects are known, revealing a tight ipsilateral link between perception and action in reacting on an external stimulus (e.g., Hommel & Prinz, 1997; Poffenberger, 1912; Simon, 1969; Simon & Rudell, 1967). In sum, there is strong evidence, at least for the 24‐ and 36‐month‐olds, that the spoon presentation during training will indeed provoke children to ipsilateral grasping, which is necessary for building up a bias.

Handedness is a well known and much‐studied factor involved in limb selection. As a working definition of handedness, we will regard it as an asymmetry property of a bistable system. Any proper definition of handedness must consist of at least two components: direction and strength. First, and most obvious, is the direction of the asymmetry: One has either a right‐hand or a left‐hand preference. From a dynamical systems point of view, we can say that this part of the definition stresses the asymmetry of the action system, that is, the fact that it has only two stationary states that differ in stability. (The special case of ambidexterity is captured by the strength component.) Second, and more subtle, the persistency with which the preferred hand is used over a wide range of tasks gives us an indication of the strength of the handedness. Handedness strength, therefore, reflects how much this asymmetry property of the action system “resists” external forces (i.e., perturbations) that drive the choice toward the nonpreferred hand. In terms of dynamical systems, handedness strength refers to the stability difference underlying the asymmetry property. More specifically, it expresses how one of the system's states is more stable than the other.

The possible functional (i.e., psychological) or structural (i.e., biological) origins of the tendency to prefer one particular hand over the other, and the way this develops with age, are not within the scope of the present paper. In fact, limb dominance is known to develop by mechanisms not yet understood in full length (Hopkins & Rönnqvist, 1998; Michel, Nelson, Babik, Campbell & Marcinowski, 2013; Scharoun & Bryden, 2014). Relevant for the present study, however, is that young children's handedness is not at all (or at least not yet) a fixed and full‐grown property of their action system. For instance, it has been reported that in the first years of life handedness fluctuates between left‐handedness, right‐handedness, and bilaterality (Carlson & Harris, 1985; Corbetta & Thelen, 1996; Fagard & Pezé, 1997; Gesell & Ames, 1947). In addition, as mentioned earlier, handedness strength is known to increase during the first 3 years of life and onward (McManus et al., 1988; Scharoun & Bryden, 2014). Consequently, we expect a difference in handedness strength between the three age groups, which might influence the results of our experiment. To determine children's handedness direction and strength, we performed a number of unimanual grasping trials without training, preceding the actual experiment.

## 2 METHOD

### 2.1 Participants

Children of three age groups participated in this experiment: Twenty‐six 14‐month‐olds, 26 24‐month‐olds, and 26 36‐month‐olds, who were all within a 4‐week range around this age. The data of 21, 19, respectively, 26 children were used for further analysis. The others were not used because of procedural or equipment errors, or because the child did not perform the task completely or refused to participate at all. The group of the 14‐month‐olds contained 14 boys and seven girls, the group of the 24‐month‐olds contained eight boys and 11 girls, and the group of the 36‐month‐olds contained 11 boys and 15 girls. Children were recruited from birth records of the city of Nijmegen. They were rewarded for their participation by means of a certificate with photograph and also received a financial compensation for travel and parking expenses. The study was conducted in accordance with the Declaration of Helsinki (1964), and written informed consent was acquired from the parents of each child.

### 2.2 Material

**Figure 1:**
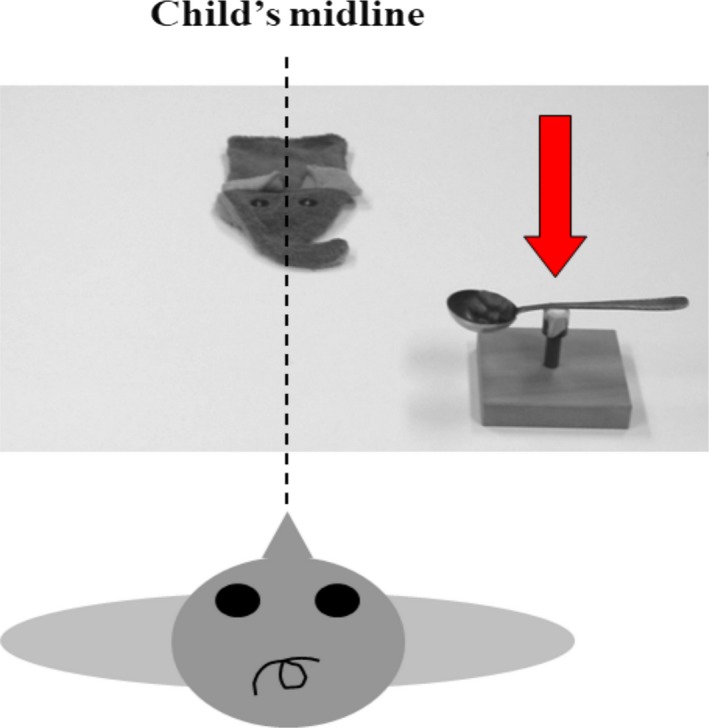
Setup of the experiment. Displayed is an example of a training trial with spoon position and handle orientation right. The (red) arrow demonstrates the direction in which the spoon is transported at the start of the trial

In the perseveration part of the experiment, we used a metal spoon, a spoon‐holder (wood and plastic), a plastic toy paprika, and a terry‐cloth hand puppet. These objects are shown in Figure 1. In the handedness part of the experiment, an additional plastic toy gnome of 7.1 cm high and about 3.8 cm in diameter was used. The spoon's handle was about 13 cm long and had an oval‐shaped bowl of about 5.5 cm by 4.5 cm, 1.5 cm deep. The relatively deep bowl of the spoon minimized control demands during transport of the paprika toward the hand puppet. This might otherwise be a potential source of difficulties, especially in the youngest‐age group. The spoon was presented on the spoon‐holder about 10 cm high above the table, making it easy to grasp. The mass of spoon plus toy paprika was 56 g, while the mass of the spoon alone was 37 g. There were three different hand puppets used: a fish, a duck, and an elephant. All three could open and close their mouth by movements of the experimenter's hand, making them especially suitable for playing a pretend‐to‐feed game.

### 2.3 Procedure and design

The experiment was performed in a quiet research room on the university campus especially equipped to accommodate children. Upon arrival, the necessary time was taken for the child to become acquainted with the room and the experimenters. After this, the child was positioned at the long end of a table, with an experimenter sitting in front of her on the other side. During the experiment, the 24‐ and 36‐month‐old children were standing on a footboard to prevent them from walking around. The 14‐month‐old children were seated on their parent's lap to assure the necessary postural stability. Before the start of each task, the children were allowed to explore the objects that were going to be used for a while. Each child was subjected to three series of trials, one handedness condition, and two perseveration conditions, which we will describe below. The handedness condition always preceded the two perseveration conditions, which were presented in a random order. In between the conditions, there was a period of pause and free play.

#### 2.3.1 Handedness condition

In this part of the experiment, children performed a number of trials in order to determine their handedness strength and handedness direction. These trials consisted of a total of nine unimanual grasping movements on objects in hemispace. In each trial, an object (spoon or gnome) was positioned and/or orientated differently relative to the child, as follows: In two subsequent trials, the toy gnome was positioned at an easy reaching distance, randomly, once in front of the child's left shoulder and once in front of the child's right shoulder. In two trials, the spoon was positioned, in a random order, in front of the child's left shoulder with its handle pointing to the left and in front of the right shoulder with its handle pointing to the right. In two trials, the spoon was positioned on the child's line of sight, once with its handle pointing to the left and once with its handle pointing to the right (in a random order). In three trials, the spoon was presented on the child's line of sight with the handle pointing toward the child. This setup enabled us to determine handedness direction and handedness strength (at least at the group level). Moreover, it provided us with a measure for hand‐use variability as a function of object position and orientation, both combined and separately.

#### 2.3.2 Perseveration conditions

In this part of the experiment, each child was subjected to two separate series of trials. During the introductory phase, an experimenter explained to the child that the animal (i.e., the hand puppet) was hungry and needed to be fed. Because it was too little to eat on its own, the child was asked to help it by using the spoon. The experimenter proceeded by putting the hand puppet around her hand and presenting it just out of reach on the child's line of sight. Each trial started by placing the spoon on the holder, also out of reach, in one of the two following position‐orientation combinations: Either in front of the child's left shoulder with the handle pointing to the left, or in front of the child's right shoulder with the handle pointing to the right, or on the child's line of sight with the handle pointing toward the child (Figure 1 for an example). After a short delay, spoon plus holder were pushed toward the child in a straight line. The child was allowed to grasp the spoon when it was within reach at her end of the table. After feeding the hand puppet, the spoon was retrieved, and a new trial was started in the same way.

**Figure 2:**
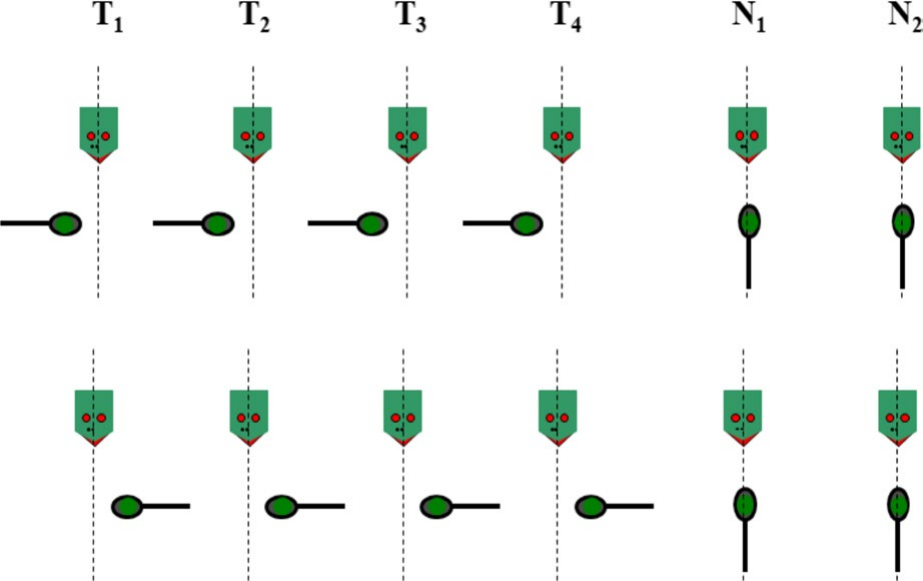
Design of the perseveration conditions. The dotted line represents the child's midline. Each condition has four training trials (*T*_1_ to *T*_4_) with the spoon either left or right, and two neutral (*N*_1_ and *N*_2_) trials where the spoon was on the child's line of sight

Each perseveration condition consisted of six subsequent spoon presentations. These conditions first established a bias of the child's left or right hand, by presenting the spoon either four times on their left side with the handle pointing to the left or four times on their right side with the handle pointing to the right, respectively (Figure 2). In the first two of these training trials (*T*_1_ and *T*_2_), the experimenter tried to “persuade” the child to use the appropriate hand by presenting the spoon somewhat more peripherally, if necessary. This was not done anymore in the third and fourth training trials (*T*_3_ and *T*_4_). The subsequent two trials (*N*_1_ and *N*_2_) were neutral trials, where the spoon was presented at midline with the handle pointing toward the child. The last two trials were performed in order to measure children's limb selection after the training set.

### 2.4 Response scoring

An observer scored the hand the child used to grasp the object with from the videotaped sessions. This was compared to a report that was scored live during the experiment by one of the experimenters. Agreement between the two was very high, and in case of mismatch, the video‐scored value was used at all times. The possible values for hand use were left, right, or both. The latter value was assigned when both hands grasped the spoon. In case of a (rare) bimanual grasping movement, the hand that grasped the spoon first was scored, even when this was altered during the feeding movement.

## 3 RESULTS AND DISCUSSION

### 3.1 Handedness condition

Using the laterally neutral trials (i.e., spoon presented on the line of sight with the handle pointing toward the child), we determined the direction of handedness for each individual child. For this, we applied the following procedure: If one particular hand was chosen more often than the other in these three trials that hand was considered to be the child's preferred hand. This is similar to what others have used to determine young children's and infants’ handedness (e.g., McCarty et al., 1999). According to this procedure, there were 11 left‐handed children and 10 right‐handed children in the group of 14‐month‐olds, in the group of 24‐month‐olds there were eight and 11, and in the group of 36‐month‐olds there were five and 21, respectively. For the subsequent analyses, the direction of the handedness of each individual child was used to transform the data from a representation in terms of left‐hand grasps and right‐hand grasps to a more general and functional form in terms of preferred‐hand use and nonpreferred‐hand use. After this, the data of both laterality groups were pooled.

In Table 1, the results of the other six trials of the handedness conditions are shown. Children's responses to these trials were used to get an idea of handedness strength at the age‐group level. From the spoon‐central trials, it becomes clear that when grasping a spoon on midline, handle orientation had a large influence on children's hand choice, especially in the two oldest‐age groups. In none of the age groups, children had an overall preference for using one of the hands (chi‐squared tests, all *p*'s >0.3). While in the two oldest‐age groups, this was due to children's (nearly) perfect mirroring of the spoon's handle, in the youngest‐age group, there was more variability in hand choice. The difference in the amount of mirroring was statistically significant, χ^2^(2, *N *=* *132) = 21.11, *p *<* *0.001. This is in accordance with McCarty et al. (1999), who found similar results for 14‐ and 19‐month‐olds. When the spoon was presented in front of the children's shoulders, as in the spoon‐lateral trials, the same age difference with respect to mirroring was found, χ^2^(2, *N *=* *132) = 9.42, *p *<* *0.01. For these trials, where the spoon's lateral position comes into play, children in the youngest‐age group displayed a clear overall preference for their preferred hand, χ^2^(1, *N *=* *42) = 6.40, *p *<* *0.05. Finally, both the youngest‐age groups showed hand‐choice variability when grasping the (orientation‐free) gnome in a lateral position. In these two age groups, there was no overall preferred hand. Contrary, the 36‐month‐olds had a (slight) overall bias toward grasping the object with their preferred hand in these trials, χ^2^(1, *N *=* *52) = 4.00, *p *<* *0.05. As a result, in none of the age groups, the gnome's lateral position was perfectly mirrored.

**Table 1:** Experimental results of the handedness condition: Proportion of mirroring object position and/or orientation and proportion of preferred‐hand use in the three tasks and the three age groups (14‐mos: *N* = 21; 24‐mos: *N* = 19; 36‐mos: *N* = 26)

Task	Mirroring object position and/or orientation	Preferred‐hand use
Gnome lateral		
14‐month‐olds	0.79	0.52
24‐month‐olds	0.83	0.50
36‐month‐olds	0.78	0.67
Spoon lateral		
14‐month‐olds	0.79	0.71
24‐month‐olds	0.96	0.54
36‐month‐olds	0.98	0.52
Spoon central		
14‐month‐olds	0.71	0.57
24‐month‐olds	0.97	0.53
36‐month‐olds	1.00	0.50

These results give rise to some general statements on handedness strength and hand‐choice variability, as a function of object position and orientation. Note again that these statements hold at the (age‐)group level. First, the overall level of variability seems to decrease with age, and seems to be highest in the youngest‐age groups. This can be gathered from the spoon trials, where the 14‐month‐olds mirrored the handle orientation significantly less compared to the older two age groups. While in the spoon‐lateral trials this was due to more preferred‐hand use, in the spoon‐central trials it was not caused by an overall hand preference. Second, handedness strength seems to be largest in the group of 36‐month‐olds, and about equal in two other age groups. Except for the spoon‐lateral trials, where the 14‐month‐olds showed an overall hand preference. Arguably this was due to the increased control demands and postural instability accompanying the more difficult lateral grasping movement. This might have made these children chose their more dexterous preferred hand more often. Finally, based on these results, it can be expected that building a short‐term bias for one of the hands in the perseveration conditions will be more difficult in the group of 14‐month‐olds. That is, the larger hand‐choice variability might lead to less “training‐hand” choices during the training trials, which will result in a weaker bias at the start of the neutral trials. In combination with the increased hand preference for lateral spoon presentations, this is expected to be largest in the training of the nonpreferred hand.

### 3.2 Perseveration conditions

Because of the transformation of the data from right‐hand use and left‐hand use to preferred‐hand use and nonpreferred‐hand use, the two perseveration conditions are now relabeled as control condition and test condition. In the control condition training was performed on the preferred hand, while in the test condition training was performed on the nonpreferred hand. In Figure 4a and 4b, the proportions of preferred‐hand use are presented for each age group in the last four trials (*T*_3_, *T*_4_, *N*_1_ and *N*_2_) of the two perseveration conditions.

The differences in handedness strength and variability we found in the handedness conditions, expectedly gave rise to different patterns of limb selection between the youngest‐age group and the two oldest‐age groups in the perseveration conditions. In the last two training trials (*T*_3_ and *T*_4_; see Figure 4), especially in the test condition, there are differences in the proportion of preferred‐hand scores between the age groups. These differences were statistically significant in the test condition, in trial *T*_3_, χ^2^(2, *N *=* *66) = 12.19, *p *<* *0.005, and trial *T*_4_, χ^2^(2, *N *=* *66) = 11.59, *p *<* *0.005, but not in the control condition, in trial *T*_3_, χ^2^(2, *N *=* *66) = 4.42, *p *=* *0.110, and trial *T*_4_, χ^2^(2, *N *=* *66) = 2.18, *p *=* *0.337. The children in the two oldest‐age groups could be trained perfectly, that is, they showed *no* preferred‐hand use in the training trails of the test condition, whereas there was only preferred‐hand use in the training trails of the control condition. On the contrary, a considerable number of 14‐month‐old children used the “non‐training” (i.e., preferred) hand in the last two training trials of test condition. As mentioned earlier, the reason for using the preferred hand more often might be related to the increased control demands and postural instability for the lateral spoon grasps.

After switching from the training trials to the neutral trials, there was a clear effect of the training on the amount of preferred‐hand use in the neutral trials. McNemar tests revealed that the observed differences in the proportion of preferred‐hand use in trial N_1_ between the two experimental conditions were statistically significant only for the two oldest‐age groups (*p *<* *0.01, for both the 24‐ and 36‐month‐olds; *p *=* *0.70 for the 14‐month‐olds). Because this undoubtedly shows that a number of these children persisted in using the hand they had been using in the preceding trials, this effect can only be interpreted as perseveration. The effect was not (significantly) present anymore in trial *N*_2_ (McNemar tests, all *p*'s >0.5).

For the children in the youngest‐age group there seemed to be no significant effect of training on the performance in the neutral trials. However, this might be due to the higher variability in their overall limb selection, especially in the training trials. Because they used the “training” hand significantly less, this might have decreased the effectiveness of the training and lead to a weaker bias. To test this hypothesis we looked at the subgroup of 14‐month‐olds that showed perfect training scores in the test condition, that is, no preferred‐hand use in the training trials of this condition. The data of this subgroup of 14 children are shown in Table 2. As was expected, this subgroup showed a statistically significant difference in the proportion of preferred‐hand use between the perseveration conditions, in trial *N*_1_ (*p *<* *0.05, McNemar test). The effect was no longer present in trial *N*_2_. Note that this group is indeed also less variable in their limb selection in the control condition.

**Table 2:** Experimental and model simulation results of the selection of 14‐month‐olds having no preferred‐hand use in the training trials of the test condition: Proportion of preferred‐hand use in the last two training trials (*T*_3_ and *T*_4_) and the two neutral trials (*N*_1_ and *N*_2_) for the control condition and test condition

Selection of 14‐mos	*T*_3_	*T*_4_	*N*_1_	*N*_2_
Experiment (*N* = 14)				
Control condition	1.00	1.00	1.00	0.86
Test condition	0.00	0.00	0.57	0.64
Simulation				
Control condition	0.96	0.97	0.90	0.85
Test condition	0.00	0.00	0.54	0.65

So although establishing a short‐term bias for the nonpreferred hand was less successful in the 14‐month‐olds, if they did have a perfect training streak their perseveration at the group level was comparable to that of the 24‐ and 36‐month‐olds. In all age groups, the perseveration effect with the preferred hand was stronger compared to the nonpreferred hand. This can readily be interpreted as multi‐causality in the action system: Since hand preference and short‐term bias both “favor” the preferred hand in the neutral trials of the control condition, this increases the likelihood for this hand to be chosen. This is not the case in the test condition, however, where there is competition between the two constraints. This leads a significant number of children to stick to the nonpreferred hand, where they would have otherwise used the (more dexterous) preferred hand.

## 4 SIMULATIONS

In the following, we will present model simulations of young children's perseverative limb selection as found in the experiment. The model that implements the internal dynamics of the limb‐selection process entails the following two coupled differential equations:

$$\tau \cdot {\dot{u}}_{P}\left(t\right)=-u_{P}\left(t\right)+h-c_{P}\cdot \sigma \left(u_{\mathrm{NP}}\right)+I_{P}\left(t\right)+n\cdot \xi \left(t\right);$$

$$\tau \cdot {\dot{u}}_{\mathrm{NP}}\left(t\right)=-u_{\mathrm{NP}}\left(t\right)+h-c_{\mathrm{NP}}\cdot \sigma \left(u_{P}\right)+I_{\mathrm{NP}}\left(t\right)+n\cdot \xi \left(t\right).$$

In the model, the time‐dependent *u* functions represent the activation functions for the limbs (sites), each of which is related to the likelihood of selecting the corresponding limb. The indices P and NP denote the preferred‐hand site and nonpreferred‐hand site, respectively. The “dot” on the activation functions on the left‐hand side of the equations symbolizes the first‐order derivative with respect to time. The parameter *h* is the resting level of the sites, to which activation will decay (with rate τ) in the absence of stimulation. The two sites are connected by mutual inhibition, σ, which is of sigmoid shape. For the strength of this inhibition the following inequality holds: *c*_P_ < *c*_NP_. It is a basic claim of the model that this difference in cross‐lateral inhibition strength represents handedness. The total input to the sites is represented by the *I* terms in the equations, which consist of both sensory input and memory input. Finally, ξ is a noise factor with strength *n*. For a more detailed description of the model see Cox and Smitsman (2018).

Both the experiment and the literature on young children's limb selection suggest an age‐related increase in handedness strength as well as an age‐related decrease of variability in unimanual grasping. To simulate our experiment in accordance with these empirical findings, we varied the corresponding parameters in our model as a function of age. Specifically, the parameters determining the strength of cross‐lateral inhibition and noise received the following numerical values: *c*_P_ = 0.5, *c*_NP_ = 3.5, *n *=* *3.3 for the 14‐month‐olds, *c*_P_ = 2.0, *c*_NP_ = 5.0, *n *=* *2.0 for the 24‐month‐olds, and *c*_P_ = 3.0, *c*_NP_ = 7.0, *n *=* *1.7 for the 36‐month‐olds. This parameter setting reflects the differences in handedness strength and variability between the age groups as we found in our experiment. These age‐related parameters and the other model parameters (τ = 3; *h *=* −*2; β = 2) were fixed throughout all simulations.

The equations were integrated using an Euler procedure in Matlab (version 6.1, The MathWorks, Inc.) on a standard PC. By taking 400 time steps of 5 ms each, we simulated 2‐s of selection‐process time, which we gathered to be a realistic estimate. All simulations are based on 500 repetitions (i.e., fictive participants), to assure convergence of the model and to obtain distributions in the results. A complete experimental procedure was simulated for each repetition: Four training trials followed by two neutral trials for each of the two perseveration conditions (i.e., control and test). The strengths of the inputs were set before the start of every trial and were active for the entire trial duration. During the training trials, sensory input to the site that corresponded to the side of the spoon presentation had strength 6.0; sensory input to the other site had strength 1.0. This difference in input strength represents the strong ipsilateral influence of object position and handle orientation on limb selection. During the neutral trials, sensory input was of equal strength (6.0) for both sites. After every trial, the strength of the memory input was updated according to the limb selection in that trial: Whenever a limb was chosen in a trial, for the next trial the memory input to the corresponding site was increased with a value of 1.2. Before and between the control and test conditions, memory input was set to zero.

**Figure 3:**
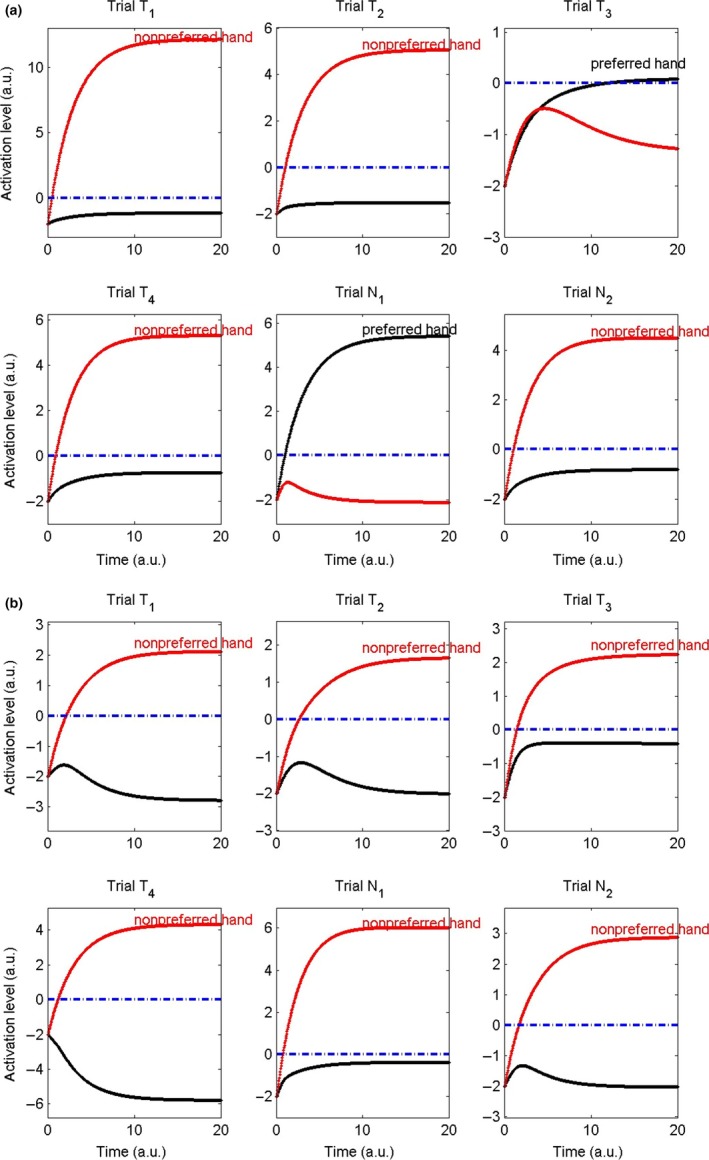
Examples of typical single‐subject simulations. (a) Simulation of a 14‐month‐old child in the test condition. Notice the variability in limb selection. (b) Simulation of a 24‐month‐old child in the test condition. Notice the perseverance in the use of the nonpreferred hand after the training trials, when the spoon is presented at midline in trial *N*_1_ and trial *N*_2_

Two typical results of a complete series of six trials are shown in Figure 3. In Figure 3a, the simulation results of a fictive 14‐month‐old in the nonpreferred hand perseveration condition (i.e., spoon presentation in trials *T*_1_ to *T*_4_ are on the side of the nonpreferred hand). In accordance with the experimental results of that age group limb selection is quite variable, in the training trials as well as in the neutral trials. The selection of the nonpreferred and preferred hand does not seem to follow a clear pattern over trials. Even more remarkable is that although the spoon is presented on the side of the nonpreferred hand, in trial *T*_3_, the subject grasps it (quite awkwardly) with the preferred hand. Figure 3b shows the results of a fictive 24‐month‐old child, also in the nonpreferred hand perseveration condition. After using the spoon with the nonpreferred hand for four times during training, the subject “sticks” to using this hand in the neutral trials where the spoon presentation is laterally neutral.

**Figure 4:**
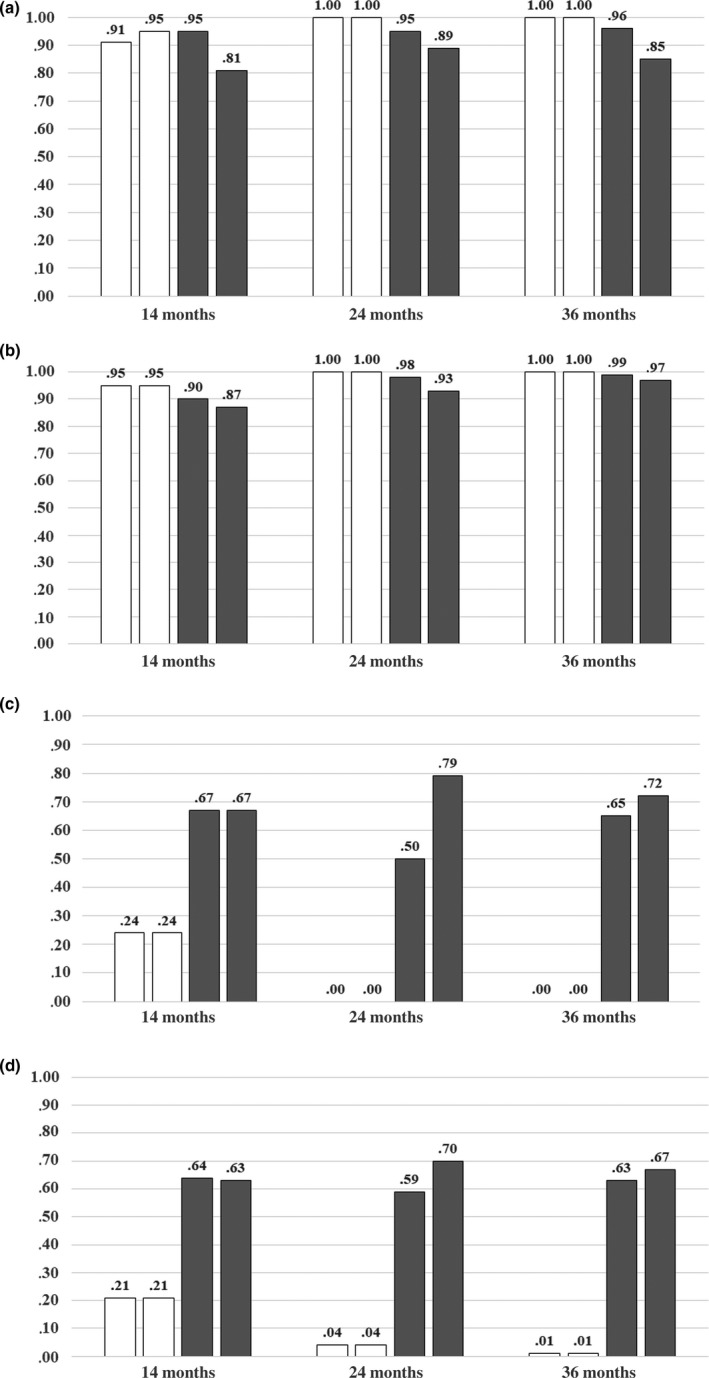
Proportion of preferred‐hand use in the last two training trials (*T*_3_ and *T*_4_) and the two neutral trials (*N*_1_ and *N*_2_) of the perseveration conditions: (a) Experimental results for the control condition, (b) Experimental results for the test condition, (c) Model simulation results for the control condition, (d) Model simulation results for the test condition

Summarizing the main finding of the experiment, which the model has to reproduce: First, the higher variability in limb selection found in the training trials of the youngest children. Second, the effect of perseveration that was found in the two oldest‐age groups. Third, the effect of perseveration that was found in a subgroup of the youngest children, filtered for 100% training effectiveness. Figure 4c and d present the statistics of the simulations for 500 runs of the model for each trial, performed separately for each age groups. Comparing Figure 4a with Figure 4c and Figure 4b with Figure 4d reveals a similar overall pattern of limb selection between the results of the simulation and the experimental results in all three age groups.

In Table 2, the statistics of the selection of simulated 14‐month‐old children with a perfect training streak in the test condition are shown. Comparing the results in Table 2 demonstrates that the model produces the same overall pattern of limb selection as were found in the experiment. Note that due to the relatively high level of noise and smaller asymmetry (parameter settings) for this age group, the model selected the nonpreferred hand in the control condition a few times, resulting in a training set which was not perfect in this subset. These minor differences between empire and simulation could surely be resolved by some further fine tuning of the parameter setting.

## 5 GENERAL DISCUSSION

The present study addressed perseverative limb selection in young children in the age of 14, 24 and 36 months. An experiment was performed in which children were provoked to grasp a spoon repeatedly with the same hand, in a series of training trials. After this, spoon presentation switched to a laterally “neutral” position and orientation. We looked at how the choice of hand for grasping the spoon within these neutral trials was influenced by the prior series of choices in the training trials. The experiment revealed that the children in the two oldest‐age groups perseverated in their choice of hand. In the youngest‐age group, this seemed not to be the case. This negative result, however, proved to be caused by the overall “quality” of their training, that is, the strength of the short‐term bias. This could be concluded after analyzing the results of a subgroup of the 14‐month‐olds, defined by having “perfect” training results. This subgroup did show a significant level of perseveration in their choice of hand. Summarizing the main results of the experiment: First, all three age groups showed some degree of perseverative limb selection. Second, the action system's recent history (i.e., the motor memory established by the training) is central to this effect. Third, the size of the effect was influenced by handedness strength, which, as an overall level of variability, influenced the effectiveness of the training.

This study adds to the conclusion that perseveration, as a general behavioral phenomenon originates from the dynamical, nonlinear, and multi‐causal nature of goal‐directed behavior (see Thelen et al., 2001). As mentioned in the introduction, this perspective on perseveration can give us valuable insight in the development of goal‐directed behavior. Our current view is that perseveration should not be understood as an intermediate developmental “stage” toward a more flexible action system, and certainly not as a developmental (end) goal. It seems to be the result (artifact, as you will) of the multi‐causal, multi‐timescale dynamics governing action selection, which is not specific to a certain age range. Under certain conditions, specifically after repeating the same action a number of times in succession, the action system will lose some of its flexibility and perseverate. Limb selection specifically does not result from either hand preference (stability) or context (flexibility) alone. It is their combination that ultimately balances the action system between optimal adaptability and optimal control.

We have brought forward a way of understanding how different components on different timescales interact over time to codetermine action selection (Cox & Smitsman, 2008). The study presented here did not only show perseveration and the effect of prior choices on limb selection, clearly establishing it as a dynamical process, it also offered a model that brings together external (i.e., spatiotemporal) and internal (i.e., subject) constraints into one concise quantitative framework. Below, we will discuss the general conclusions following from these results, in terms of the dynamic model, and what they suggest about action planning and action control in goal‐directed behavior in general and in young children in particular.

### 5.1 Dynamic model

One of the main goals of this study was to extend an existing model of limb selection (Cox & Smitsman, 2018) from adults to children in the age of 1–3 years. More in particular, we intended to incorporate into the limb‐selection model, the developmental changes in handedness that are known from the literature and that were (partially) reproduced in the handedness condition of this study. To demonstrate how these changes influence the selection process, the model was used to simulate a limb‐selection perseveration experiment.

Two main parameters implemented these developmental changes in handedness: the noise level and the (difference in) inhibition strength. The noise level reflected the overall variability in hand use. It determined the level of spontaneous activation of one of the two sites, increasing the likelihood of the corresponding hand being selected for performing the reach. This random force has its influence in the dynamics of the selection process next to the sensory and memory input. In the model, handedness (strength) was incorporated by the difference in the strength of the inhibition between the sites. This was already introduced in the initial version of the model (Cox & Smitsman, 2018). In the present context, however, differences in inhibition strength have a developmental meaning. The difference in handedness strength between the age groups in our experiment was reflected by the difference in inhibition strength, which was suggested to increase with age (especially the oldest‐age group seemed to have a stronger hand preference than the other two).

Motor memory was treated as an input source that itself builds over an intermediate timescale with respect to the dynamics of the limb‐selection process. Dynamic preshaping of the action‐selection field by the motor history of the system (memory trace) has already been introduced in the dynamic field theory (Dineva & Schöner, 2018; Erlhagen & Schöner, 2002; Thelen et al., 2001; Schöner & Dineva, 2007; see also Ibáñez‐Gijón & Jacobs, 2012). In contrast, handedness (as an overt behavioral preference) was treated as an asymmetry of the dynamical system governing limb selection. A larger asymmetry in the governing dynamics, that is, a larger difference in inhibition strength, reflects a stronger handedness. Incorporating structural or functional asymmetries (i.e. long‐term biases) as an integral part of the dynamics is a novel aspect for this type of models. As such, the present model continues on the path of earlier dynamic models, in particular, the dynamical field model developed for perseverative reaching in the A‐not‐B task, by showing how constraints related to long‐term biases of an action system can be dealt with on an equal footing with other type of constraints.

### 5.2 Action planning and action control

Often it is assumed that action planning occurs in an “offline” phase, generally preceding the actual performance of the planned action (see e.g. Hommel, 2003). In this phase, a sketch of the required actions and the expected outcomes are constructed under the influence of high‐level cognitive processes, involving intentions, action‐effect relationships, heuristics, and strategies. Detailed sensory information is usually not assumed to be present yet during the planning phase. Sensory information is supposed to be monitored and dealt with by lower‐level “online” processes during the control phase of action. By then the action planning is completed, and the process of action control guides the execution of the planned action, for instance by means of error correction mechanisms.

However, action planning is all about prospective control of behavior (Bertenthal & Clifton, 1998; Gibson & Pick, 2000; Smitsman, 2001; Von Hofsten, 2003). In other words, action planning is about being adaptive in a changing environment in order to be goal directed and stay goal directed. This means that action planning has to be as online and attuned to the environment as action control. Planning and control must be temporally and functionally integrated to a large extent for behavior to be effective and efficient (Spencer & Schöner, 2003). In fact, action planning and action control are likely referring to a single overarching action‐selection process in which prospective and reactive mechanisms merge. In order to adequately and flexibly deal with the dynamical aspects of behavior, imposed for example, by changing or ambiguous perceptual information or by coordinative action between partners during interpersonal interaction, action selection has to be an ongoing and dynamical process. We believe that sensory information, intention, as well as functional and structural properties of the action system, such as preferences and the like, continuously interact in real time and that behavior emerges from these interactions. Moreover, the proposed action‐selection process is part of the early phases as well as the execution phase of action, in fact of each phase one wishes to distinguish in behavior. The view of action selection presented in this paper, in particular, the merging of long‐term (offline) preferences with short‐term (online) sensory information, provides a framework for thinking about this (see also Cox & Smitsman, 2008).

As a final consideration, we note that it is well known that a certain level of prospective control of action is already visible in neonates (Lee, 2006; Von Hofsten, 2003). This has been very elegantly demonstrated, for instance, by studies on infant sucking behavior (Craig, Grealy & Lee, 2000; Craig & Lee, 1999). These, and other studies alike, suggest that the new‐born action system is functionally and/or structurally equipped to combine different sensory, motor and (early) cognitive processes in order to engage in goal‐directed behavior. Considering this, we would like to promote a perspective on cognitive and action development that addresses how different constraints on different timescales are intertwined. Behavioral development, then, results from changes in the relative contribution all these constraints have in the action‐selection process.
